# Notice of Republication: Simulating Cortical Development as a Self Constructing Process: A Novel Multi-Scale Approach Combining Molecular and Physical Aspects

**DOI:** 10.1371/journal.pcbi.1006747

**Published:** 2019-01-23

**Authors:** The PLOS Computational Biology Staff

This article was republished on November 28^th^, 2018, to correct an outdated link in the Funding statement. The link has been replaced but otherwise the wording of the Funding statement remains the same.
